# Employing innovative evidence-backed community processes for maternal health services by Dalit women

**DOI:** 10.1186/s12939-022-01776-4

**Published:** 2022-11-21

**Authors:** Sathyasree Goswami, Edward Premdas Pinto

**Affiliations:** 1 Tata Institute of Social Sciences, Deonar, Sion-Trombay Road, Mumbai, India; 2 School of Development, Azim Premji University, Burugunte Village, Sarjapura Campus, Bangalore, India

**Keywords:** Dignity and health, Social justice, Social citizenship, Social accountability process, Dalit women and accountability, Human right to health, Politics of health, Maternal health, Caste and Social accountability

## Abstract

**Background:**

Health care services express social and structural inequalities, especially for Dalits and women, due to the indignity and discrimination experienced in health care facilities. Jagrutha Mahila Sanghatane (JMS), a grass-roots organization led by neo-literate Dalit women in rural Karnataka in India, adopted a human rights-based social accountability (SA) approach to address discrimination and dignity in accessing maternal health services. This approach integrated community-based evidence with multi-pronged and multi-level accountability processes with their goal of socio-political empowerment.

**Methods:**

The methodological approach is qualitative and uses document analysis, including thematic and content analysis, in-depth group discussions with the campaign leaders, participant observation and interviews with the community health workers.

**Results:**

JMS embedded the practice and processes of SA in the politics of empowerment which was central to addressing the structural issues of discrimination and social exclusion faced by Dalit women. The human rights perspective and the pathway of conscientize-organize-struggle provided by the Dalit liberation leader, Dr B. R. Ambedkar, facilitated the organization to conceptualize SA as a process of claiming dignity and justice for Dalit women. Integrating the evidence generation and its deployment into the community campaign cycles, Dalit women could use the accountability process for intensifying mobilization and empowerment. The cumulative impact of the community enquiry relentlessly pursued through the framework of a campaign brought changes in several aspects of primary health care and specific dimensions of maternal health care. Community ownership of the SA process, participation and empowerment were integral to the generation, synthesis and deploying of evidence. Deploying evidence in multiple forms, both horizontally with the communities and vertically with the authorities deepened communities' mobilization and intensified Dalit women's negotiating power with the authorities. The iterative and persistent process of SA provides insights into re-articulating SA beyond the usual recognition of outputs such as report cards into the politics of meaning-making by the mobilized community of the marginalized. The community-based organization posited the SA itself as the process of resistance to structural injustice and as an avenue or their empowerment.

**Conclusions:**

For marginalized communities, the SA process has the potential to be a tool for their empowerment in addressing structural power inequities. For such a repositioning of SA, it is critical to focus not only on the technicality of generating evidence but also on the framework driving such a process, the mode of evidence generation and deployment, and integration into the organizational strategy. Such a process can be equally empowering, efficient in addressing the systemic challenges of increasing marginalized community's access to health care services, and valuable in sustaining those changes. The analysis of the strategies of JMS offers significant insights for researchers and practitioners working on SA and maternal health to re-articulate SA from the point of politics of empowerment of the marginalized communities.

## Introduction

For Dalit women, indignity and discrimination experienced in health care facilities are an experience of everyday lived reality and an expression of the overarching systemic oppression they face in public spaces and institutions. Discrimination experienced by Dalit women is pervasive in the private spaces of their homes, public spaces of communities and neighborhoods, and the formal spaces of governance and public institutions [1]. Despite the constitutional abolition of untouchability in India [2], such a practice has continued unabated against Dalits in Indian society because of their caste. Such a cultural practice legitimizing segregation of physical resources and avoidance of physical proximity, based on the concept of purity and pollution, is manifested in various forms such as the exclusion of Dalits from the social functions (e.g., marriage, festivals, inter-dining) of all non- Dalits, use of separate teacups in teashops, and cultural sanctions on Dalits not to enter temples. In the case of health care, caste-based discrimination unfolds in several ways, primarily through the attitudes and practices of health personnel who primarily belong to the non-Dalit castes, directly impeding Dalit women's access to health services. For example, Auxiliary Nurse Midwives (ANM), the front-line health workers belonging to non-Dalit castes, do not visit Dalit houses and hesitate to touch pregnant women or children as it involves physical proximity. Further, being financially resourceless, Dalits cannot afford private health care services [3].

Therefore, in examining Dalit women's health, there is a need to move beyond pathology-based thinking on health (as a disease) and contextual conceptualization concerning the social production and structural determinants of health and wellbeing [4–10]. Caste and gender-based discrimination, its entrenchment in the health care system and its impact on poor health outcomes are very well acknowledged in literature [11, 12]. The discourses on health and human rights note intersections of medical care with the organization of public health, ethics, and social hierarchies based on gender, race, caste, patriarchy, ethnicity, and disability [8, 9, 13, 14]. The historical narratives of Dalits and contemporary research have alluded to strong linkages between institutional entrenchment of caste-based social exclusion and unequal health outcomes [11, 12, 15, 16]. Moreover, critical public health literature argues that health care services and institutions are expressions of social and structural inequalities [17]. These studies, however, do not offer any pathways or strategies to address structural discrimination. There is a need to learn lessons from the sustained accountability strategies of the historically oppressed communities for demanding health rights. Their approach to blending the human rights and constitutional perspectives with the collective strategies and actions for addressing structural discrimination holds significance in such a venture.

In this paper, we aim to document and analyze the multi-pronged and multi-level accountability processes of Jagrutha Mahila Sanghatane (JMS) and its innovations in using community-based evidence to address systemic caste and gender-based inequities in maternal health care. JMS is a Dalit women's community-based organization (CBO) led by neo-literate Dalit women in rural Karnataka (India). It employs a human rights-based framework to steer their socio-political empowerment. Therefore, we focus on understanding the impact of social accountability (SA) processes powered by human rights perspectives employed by this organization to address caste and gender-based discrimination in public health care services and the specific issue discussed in this paper, viz. reproductive and maternal health care. In such an analysis, this paper is mainly guided by the following research questions: One, how did neo-literate Dalit women agricultural laborer’s collective in rural India engage with addressing caste and gender-based violence conceptualize SA?; two, how did the collective prioritize maternal health issue for SA in the organization?; three, what insights could accountability practitioners and researchers draw from the strategies of integrating community-based evidence on reproductive- maternal health with the socio-political processes of empowerment.

### Conceptual framework

Several accountability practitioners and organizations have employed community-based evidence to augment the outcomes of such SA processes. These are known by several names, including community monitoring, community-based monitoring and planning, community enquiry (CE), and community action for health [18–21]. This paper refers to them as CE. The SA literature also classifies these initiatives based on their approaches as tactical or strategic. The tactical SA initiatives are said to be bounded, localized and revolve around supplying information to the communities. The strategic approaches tend to bolster government capacity to respond to community voice [22].

Notably, SA initiatives often are confined to the health service delivery located in the health centres and pay less attention to the processes of social exclusion embedded in the health system functioning. In our understanding, such SA processes need to go beyond merely being deployed as tactical tools to increase access to health services and﻿ primarily be foregrounded as strategic tools seeking to resist and dismantle the structures that create such inequities and disempowerment. In the latter, there is a more significant potential to strengthen social citizenship that reinforces equality and dignity [23]. Understanding the historical and structural processes of social exclusion Dalit women experienced in accessing health services has prompted us to draw a three-pronged theoretical perspective to analyze the evidence-based SA processes in this paper.

The human rights framework for health care, articulated in the International Covenant on Economic, Social and Cultural Rights (ICESCR), sets the parameter for health as ‘the highest attainable standard’ [13, 24]. The General Comment 14 also expresses its operational dimensions regarding availability, accessibility, affordability and quality of health services. It institutes them as the central tenets for gauging the fulfilment of human rights in health [14, 25]. Two, the theory of social citizenship, propounded by Marshal and Bottomore, complements this by positioning economic, social, and cultural rights (referred to as social rights) as integral to realizing and achieving social citizenship on par with civil and political rights [23]. Three, the understanding of intersectionality prompts us to take note of the triple burden of Dalit women based on their caste, gender, and class as Dalit women belonging to the daily-wage labour class in the informal sector [26].

We postulate, therefore, maternal health care as a social right integral to Dalit women's personhood and dignity and hence integral to the realization of their citizenship. While doing so, we also posit SA in this paper as an iterative and persistent process having the potential to confront social exclusion in public and institutional spaces towards establishing the citizenship of the oppressed communities. The case of the maternal health rights campaign by Dalit women discussed in this paper illustrates the process of claiming citizenship through the social right to health care that includes reproductive and maternal health.

### Context of study

#### The cultural region of hyderabad Karnataka

**Fig. 1:**
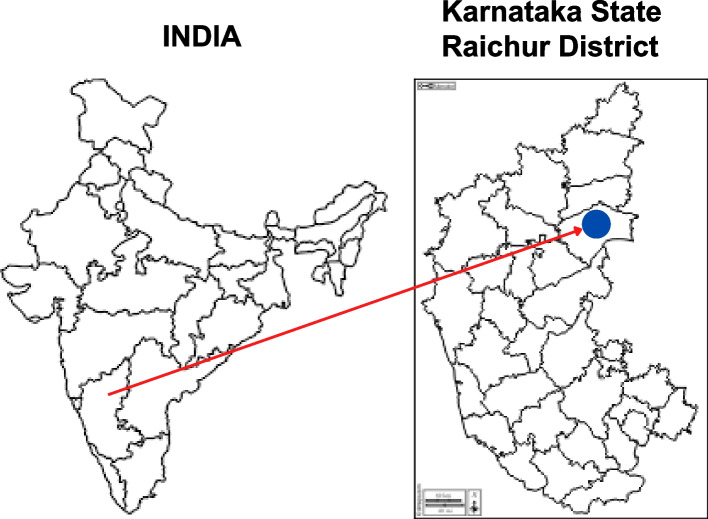
Location of Raichur in India

This study is located in the Raichur district of Karnataka state in India, sharing the borders with two other prominent South Indian states, viz. Andhra Pradesh and Telangana, and form an integral part of the cultural region of ‘Hyderabad Karnataka’ (see Fig. 1). The overall literacy rate in this district of 19,28,812 population is 59.56 percent. It is one of the lowest in the state, consisting of thirty-one districts. Dalits, constitutionally categorized as Scheduled Castes (SC), constitute 20.79 per cent of the population and have a literacy rate of 45 per cent, of which male and female literacy is 61 percent and 39 percent, respectively [27]. The 27.2 percent of cultivators in the district predominantly represent the land-owning upper caste peasantry. Dalit households are landless, working either as daily wage agricultural laborers or tenant farmers, still compelled to show allegiance to these landlords.

The dominant narratives of general backwardness of the region or the marginalization of Dalits camouflage the extreme physical, sexual and psychological violence experienced by Dalit women on account of caste and gender. A sizable proportion of the 42.5 percent agricultural workforce comprises Dalit women [27]. The untouchability and other forms of caste-based discrimination that persist have a close relationship with these land and economic relations [28]. The outcome of such caste-based exploitation is the denial of subjectivity to Dalits by depriving them of dignity and personhood [29]. This region accounted for frequent violence and atrocities on Dalit women for issues related to inter-caste interface that included love and marriage and sharing common spaces (wells, water sources, hotels, transport). Some ethnographic studies recorded the systemic violence experienced by Dalit women based on their gender and caste [30]. Given such a context, it is unsurprising that the Raichur district is relegated to a low status in the Karnataka Human Development Index [31, 32] and the Gram Panchayat Human Development Index 2015 [33]. Such backwardness was also affirmed by a special committee constituted by the Government of Karnataka [34]. Subsequently, the region was granted special status under article 371 of the Constitution of India, entitling it to special economic packages and policy, privileging the people of this region in education and occupation [35].

### Jagrutha Mahila Sanghatane (JMS)

JMS was initiated in 1999 through an extensive social mobilization process to address caste and gender-based violence against Dalit women whose primary occupation was agriculture labour. The understanding and analysis of the triple oppression of Dalit women as Dalits, agricultural laborers, and women, referring to caste, class and gender, respectively, laid the foundation for the evolution of this Dalit women's collective in its formative years [36, 37]. The collective draws inspiration from Dr B. R. Ambedkar, the icon of Dalit liberation in India and a legal stalwart who framed the Constitution of India. It follows the three-pronged pathway he laid down—organize, educate (conscientize) and struggle (claim rights)—for the liberation of Dalits. It is a membership-based grass-roots organization that currently consists of 4600 women [38], drawn from fifty villages of 25 Gram Panchayats (GP), intersecting with 10 Primary Health Centres (PHC). GP is the lowest administrative institution in India for a 10,000 population, and PHC is the basic unit of organizing health care services in rural areas catering on an average to a 30,000 population. JMS office is in 'Pothnal' village, seventy kilometers from the Raichur district headquarters and is close to four of the seven talukas (subdistricts, elsewhere also known as blocks) of the district, viz. Manvi, Sindhanur, Maski and Sirwar. JMS, as a women's collective, has a circular model of organizational structure. The group of conveners, known as *sanchalana samiti*, comprises senior Dalit- women leaders and two male associates. They have been part of the organization since its inception in 1999. As a group of senior leaders, it is the key driving force behind the strategies of social interventions employed by the organization. The women's collective is composed of village units of women. It is organized either as self-help groups (a group of 15–20 women in the village who meet every week) or as labour groups (having 30–40 members who primarily focus on accessing the right to one hundred days of work that is implemented in India by law).

Two representatives from each village unit are nominated to form the Coordination Committee, or *karyakartha samiti* of the organization. It is the central body that governs the collective. The leadership and majority of the women members of the collective belong to the Dalit-*Madiga* community. *Madiga* is the most disadvantaged social group within the large canopy of marginalized community that has identified itself as Dalits. However, in recent years, JMS has opened its doors to other daily wage women-workers from non-Dalit and Muslim communities too. The leadership of the organization is supported by mentors who have been associated with the collective for a long time [37].

JMS constituency encompasses ten of 56 PHCs in the district of Raichur. Of these, seven PHCs have been the focus of SA processes for maternal health rights, of which five are enlisted as 24 × 7 PHCs [39, 40].

## Methods

The methodological approach to the inquiry in this paper is qualitative. It uses four qualitative methods, viz. document analysis which includes thematic and content analysis, in-depth group discussions with the group of campaign leaders (4), key informant interviews (8), and participant observation of the campaign processes.

### Document analysis

The qualitative document analysis method entails a four-stage process of finding, selecting, appraising and synthesizing [41]. However, typologies of qualitative and documentary data analysis vary [42]. Consent was obtained from the President of JMS to access archives. The documents consisted of four files having 119 documents (see Table 1), two printed books [36, 43], and digital documents (see Table 2). The researchers obtained photocopies of the physical documents and soft copies of the digital documents.

**Table 1:** Year-wise and thematic classification of physical documents

Year	Documents	Number
2015	• Petitions to authorities	2
2016	• Petitions to authorities (4) • Press-release (1)	5
2017	• Press-release (1) • Collated report of community enquiry (1) • PHC-wise report card printed banners (7) • Pamphlets/leaflets (2)	11
2018	• Government Orders and documents issued by various authorities in response to petitions (4) • Leaflet summarizing demands (1) • Report cards printed as banners (7) • People’s Health Manifesto for Assembly Elections – pamphlet (1) • Women’s Gram Sabha Minutes of Pothnal GP (1) • Media coverage clippings (1)	14
2019	• Petition related to Maternal health services given to various health systems related authorities (33) • Petition related to Nutrition and Integrated Child Development Scheme (ICDS) given to Women and Child Department authorities (8) • Pamphlets summarizing maternal health CE reports and demands (2) • Case-study on complicated delivery (1) • File with printed info-graphic reports on ante natal care (ANC) and post-natal care (PNC) services (1file with forty sheets) • Petition related to other social rights – sanitation, water, housing, work (2) • Police permission letter to hold rally (1) • Pamphlet on public protest (1) • Application to visit Chief Minister (CM), acknowledgment and pass, a memorandum submitted to CM (3) • Press-brief (1, released to fifteen newspapers) • Media coverage clippings (Kannada 4, English Daily 4)	61
2020 & 2021^a^	• Petition related to Maternal health services given to various health systems related authorities (15) • Petition related to other social rights – sanitation, water, housing, work (1) • People’s Health Manifesto – GP elections (1) • Women’s health and patient rights (1) • Government Orders and documents issued by various authorities in response to petitions (1) • Pamphlet on Public Health Dialogue at District Health Officer (DHO) office (1) • Media coverage/press clippings (Kannada 7)	26
	**Total**	**119**

^a^Updated: 20 December 2021 The entire data collection and analysis process posed a challenge to the authors due to the insider–outsider role in the campaign. The authors had to be conscious of this overlap in the analysis and their perceptions and biases to minimize them in the analysis

**Table 2:** The digital data relevant to JMS and the maternal health campaign

Digital document	Source	Reference code
JMS website (old)	www.jmschiguru.org	JMSWebsite_old
JMS website (new)	www.jmschiguru.com	JMSwebsite_new
Documentary film on JMS*, hejjegalu*	https://www.youtube.com/watch?v=zDeoA7GJDhQ&feature=youtu.be	JMSFilm
Social media accounts like Facebook	https://www.facebook.com/jms.chiguru.3	JMSChiguru_FB
twitter of JMS	https://twitter.com/DalitWomenUnite	JMS_twitter
A short video on the JMS maternal health campaign	https://www.youtube.com/watch?v=NzXoNl1uOkU	JMS_MHFilm
Compiled reports of the campaign for 2018, 2019, 2020/21	JMS Digital Archives	MHReport_2018 MHReport_2019 MHReport_2020
JMS organizational evaluation report 2011 (one document)	JMS Digital Archives	JMSReview_2011

### In-depth group discussions

*(n* = *4)****:*** Semi-structured in-depth group discussions (IGD) were held with the core group of JMS conveners, who are also campaign leaders. All four IGDs were held in person in 2020 between February and August months. Four key women leaders and two male associates participated in these discussions held in the JMS office. These explored reflective learning from the campaign processes, reconstructing the sequence of the campaign, challenges and listing tangible changes in health service provisioning in PHCs and villages where CE was held. As the researchers are also probono mentors, some key challenges were often posed as questions for problem-solving. For example: 'Petition is given to the medical officer (MO) to have adequate stock of medicines and stop prescribing to private medical stores; the MO has been transferred, and a new appointment has not occurred for three months. What should we do?' The researchers had to consciously park such questions to be taken up after the group discussion.

### Key informant interviews

*(n* = *8):* Eight semi-structured key informant interviews (KIIs) were held to explore the community-level challenges, community responses and the perception of health care providers. Four were held telephonically during the COVID-19 pandemic due to travel restrictions, and four were held in person. Four interviews with campaign leaders, including two women leaders and two male associates, focused on the organizational vision, dynamics and strategies and how maternal health became a priority in the organization. These were recorded with the consent of the respondents and were transcribed.

The second set of four interviews was held with the community health workers working with the health department, known as accredited social health activists (ASHAs). Since 2011, these ASHAs have been actively working in four of the 7 PHCs that are part of the maternal health campaign. The respondent ASHAs belong to the communities where the maternal health campaign was held. Owing to the discomfort of ASHAs at digitally recording these conversations, these interviews were not recorded.

### Participant observation

The authors were part of the process of the maternal health rights campaign, its conceptualization and design in 2017 and its subsequent cycle, and from time to time, they provided strategic inputs for the campaign. Besides providing input to the local artist for translating data into creative local cartoons (2018), they also took part in some events such as the village march (January 2018), public health dialogue at Sindhanur Taluka Hospital (2019), capacity building of women leaders on using the digital app (2019). They participated in the campaign review meetings of the organization in 2018 and 2019.

This process of accessing documents and verifying the information was further supported by a literate programme coordinator at JMS managing its documentation and digital platforms. Several telephonic calls and personal conversations over emails helped organize the documents in chronological sequence and clarify any discrepancy that was spotted.

The entire data collection and analysis process posed a challenge to the authors due to the insider–outsider role in the campaign. The authors had to be conscious of this overlap in the analysis and their perceptions and biases to minimize them in the analysis.

### Data analysis

The hard-copy files were first organized year-wise between 2015–2021. Within each year, the documents were then sequentially arranged according to the dates; in a few documents, the dates were confusing, and they were clarified telephonically with the programme coordinator. After reading the content, thematic categorizing of the documents followed—petitions, 'received copies' (a copy of the petitions signed by an appropriate authority with a seal as acknowledgement), press releases, pamphlets, government orders/circulars, pamphlets, and press clippings. The petitions that formed the bulk of the documents were then segregated into two categories—maternal health services and those with other social rights demands that included maternal health services. After the segregation of the documents and sequencing of them, it became apparent that the campaign did not strictly follow the calendar year and often continued to the subsequent calendar year. The annual cycle of the campaign would start in the summer (April), gain momentum after peak summer months (June) and continue till February/March of the following year (see Table 3). For the convenience of analyzing the typology of documents, the calendar year is used as the parameter.

**Table 3:** Cycles of maternal health campaign

Cycle of maternal health campaign	Years	Method of community enquiry	Issues emerged and focused on the campaign
Cycle 1	2015	Anecdotal instances of violations	Disfunctional VHNDs, women accessing private providers Health Care expenditure in private hospitals
	2016	Anecdotal instances of violations	Disfunctional VHNDs, women accessing Private providers Health Care expenditure in private hospitals
Cycle 2	2017/18	Pictorial (visual) tools on Dalit women’s access to maternal health services Documentation of Private Health Care issues	Provision of ANC & PNC services at the community level Regular conduct of VHNDs Adequate staff and care in PHCs Special facilities such as sonography at first referral units (FRU)
	2018/19	Pictorial (visual) tools on Dalit women’s access to maternal health services Documentation of Private Health Care issues	Provision of ANC -PNC services at the community level Regular conduct of VHNDs Adequate staff and care in PHCs Special facilities such as sonography at FRUs
Cycle 3	2019/20	Pictorial (visual) images used App-based tool for collation and analysis	Provision of ANC & PNC services at the community level Regular conduct of VHNDs Adequate staff and care in PHCs Special facilities such as sonography at FRUs
	2020/21	Pictorial (visual) images used App-based tool for collation and analysis	Provision of ANC & PNC services at the community level Regular conduct of VHNDs Adequate staff and care in PHCs Special facilities such as sonography at first referral units (FRUs)

Both authors analyzed the content of the documents and created a framework for thematic and content analysis. The first author, familiar with conversational Kannada, facilitated the key informant interviews. The second author, versatile in Kannada (the official language of Karnataka state), especially with the culturally distinct variation spoken in Raichur district, analyzed the documents in the Kannada language, facilitated the in-depth group discussions and transcribed the recorded data. The authors jointly held meetings to do the content and thematic analysis to identify themes and processes of the maternal health rights campaign.

### Ethical concerns

JMS is a collective led by a group of neo-literate Dalit women supported by some male activists from the community who are members of the core group of conveners. The issue of ethical concerns stems from the outsider-insider relationship of authors with this organization. The authors are associated with the organization as probono mentors, and this relationship is unrelated to any financial transactions. They support the organization in strategizing and facilitating JMS' work to an external audience and provide intellectual time voluntarily to support the organization. Though they are not the decision-makers in the organization, they enjoy the status of being insiders. The purpose of writing this paper was explained to the sanchalana samiti as primarily communicating to the academic world about its accountability processes to learn from their interventions. The authors also clarified that any financial incentive was not driving them, nor was it financed by any project. They reiterated that the paper intended to disseminate their campaign's learning and build future contacts for the organization.

Written consent is given by the organization's leadership to write the paper and to access necessary data in the form of documents, files, images or access to field activists for collecting required information. Further, general oral consent is obtained for all the group discussions with the core group and other field workers. They explained that all names would be anonymized in the paper. General consent was obtained to use the organization's name in the paper wherever required. The authors offered to place all files accessed back in the archives without disturbing their order or misplacing any document and that JMS would be acknowledged in the paper. The authors assured the organization's leadership that the files and data obtained in the soft copies would be kept confidential and used only for the purpose of this paper. Though the authors closely followed the processes of the campaign, it was also clarified that in case any doubt arose in interpreting the information, it would be clarified with the concerned persons in the organization.

When drafting this paper, one of the women leaders, a central figure in these SA processes described in this paper, died due to health-related complications. She was also part of the group discussions. The president of JMS agreed that the authors could use her input in the paper.

## Results

The conversations with women leaders and the analysis of the written and visual documents provided some profound insights into the evolution of JMS as a community-led organization of Dalit women agricultural laborers. In the following sections, we analyze the trajectories of this collective engaging with evidence to demand responsiveness aimed towards realizing better maternal health care for Dalit women and, in turn, strengthening the primary health care system. Keeping the research questions in mind, we examined the community processes of JMS stretched over six years (2015–2021) of engaging with maternal health using community evidence as the fulcrum of such a process. Our analysis of the community processes of SA in maternal health provides insights into the thought processes and phases of SA practice in maternal health. These include—how the organization has conceptualized SA in maternal health and visualized its practice; how it has institutionalized such a practice as an organizational strategy; and finally, what changes and challenges they perceive. The practitioners of accountability would immensely gain from how a neo-literate Dalit women’s contributions to internalizing, interpreting and adapting the SA perspectives to their local context and politics.

### Conceptualization and dynamic approach to SA and its politics

What does SA mean to a collective of Dalit women led by a group of neo-literate women leaders? A solid reference to this question is found in the fundamental motif articulated in the vision statement of JMS. It focuses on Dalit women’s dignity as quintessential to its politics and uses campaigns as innovative pathways of mobilizing the community to negotiate power to address the issue of dignity.

#### The centrality of dignity and empowerment

The politics and strategies of JMS focused on addressing the structural issues of caste and gender-based violence against Dalit women, and the leadership considers such a blend as ‘the key stone’ in the formation of the collective (JMSwebsite_new). The politics of empowerment and dignity echo through the organization's various strategies and the issues it has taken up over the years. The historic call given by the icon of Dalit liberation in India, Dr B. R. Ambedkar, i.e., 'organize, educate, and agitate' has been the paradigm within which JMS conceptualized and strategized its politics of empowerment. The organization has popularized this paradigm of empowerment as 'conscientize, collectivize and struggle [fight]'. The songs and slogans that the organization uses sharply express this perspective as 'educate, agitate, and fight', resonating with the local idiom. This three-pronged articulation encapsulates for Dalit women the essence of their politics of empowerment as claiming dignity and justice. It also provides them with the raison d'être for all the entailing processes, including negotiating power, confronting discrimination, challenging the apathy of the justice and investigative institutions, and claiming legitimate spaces in public institutions [43].

To this grass-roots organization, representing a community that has faced systemic injustices historically in the oppressive caste system, and for Dalit women facing the triple burden of caste, class and patriarchy, the centrality of dignity and justice has an overwhelming and defining significance. The women members privileged dignity over all tangible outcomes and located SA strategies for claiming public spaces and access to services as the quest for reclaiming dignity [43].

#### Community campaign as a lever of mobilization for SA

Along with gaining clarity on the issue of dignity, JMS leadership considers it essential to visualize the mobilization of the community for claiming rights. This process is integrated into the community campaigns and represents the primary organizational strategy for demanding rights and entitlements. Such a visualization entails using the human rights idioms of violations to intensify this mobilization, building a collective force and consistent interface with the public administration to mount a collective pressure for eliciting responsiveness. The organization has conceptualized and articulated community campaigns as strategic SA tools to address various aspects of systemic issues that marginalize them and exclude them from experiencing their human right to dignity and citizenship. Such an approach, we found, has led to meaningfully interweaving the framework of human rights that forefronts the equality and dignity of everyone into the SA strategies of the organization. This also resonates with the general notion of considering campaigns as effective ways of public communication to influence public knowledge, attitudes, and behavior toward the goal of social change [44].

Currently, JMS has organized its community interventions around six key campaigns addressing specific rights. They include the right to work and livelihood, right to food and nutrition, right to health and maternal health, Dalit women's right to dignity, citizen-rights campaign, and right to social security. These have been used to mobilize communities and organize/collectivize Dalit women agricultural laborers. The long-term strategy of addressing these issues is through campaigns that the collective of women considers 'the levers of mobilization and empowerment of Dalit women' [37]. Such thematic campaigns have origins in the individual and collective experiences of exclusion and discrimination faced by Dalit women.

A campaign cycle entails a series of interrelated steps that include community-level deliberations, documentation of violations and service deficiencies, formation of various community delegations for representation before various authorities, drafting a memorandum on critical issues, and negotiations with authorities. Such a representation is done at various levels of public authorities, from the GP at the village level to the block and district level officers. Representation is also done before the state-level authorities through the various social movement alliances in which JMS is active. Such representations, employed as measures for exerting pressure on the administrative authorities, are iterative and, when needed, are deployed simultaneously and hence are not linear or sequential. In practice, such a mobilization and demanding accountability process correspond closely with the idea of 'social citizenship' [23].

The campaign gains momentum at different intervals quite organically within its cycle. Each campaign cycle takes a year, starting with the summer of one year, and closing at the end of spring in the next year. It then spirals into the cycle of the subsequent year, building on the momentum of the previous one. Campaigns have their peaks and nadirs in the life cycle of the organization as well. Some campaigns have continuously kept the issues of exclusion and marginalization alive in public discourses, such as Dalit women's human rights.

In contrast, some other campaigns, such as health and nutrition, pick up the pace gradually, and hold a low profile for some time when campaigns such as right to work become prominent in the organization. However, they are never abandoned, and they resurface later at an appropriate time, resonating with the community’s priority issues. The organization considers these campaigns 'levers of mobilization and empowerment' [37].

A sustained conscientization process and a sharpened articulation of community issues in the language of human rights and violations for negotiating with the administrative and societal institutions are at the core of these campaigns. The SA for maternal health is part of this campaign strategy. It has organically evolved as integral to the organization's politics of empowerment for claiming dignity and justice.

### Incorporating maternal health into the organizational strategy

We examined how JMS involved itself in building community cooperation for the maternal health rights campaign. The ability of JMS to link the unavailability of services and the women's growing indebtedness due to health care expenditure to the systemic exclusion and express them in human rights and accountability language drew our special attention.

The analysis of SA around maternal health illustrates how the issue of maternal health gained momentum in the constituency of this grass-roots organization. Through internalizing the human rights understanding, the organization steadily grew into realizing that Dalit women need to conscientize themselves with maternal health rights, organize the community around these rights and build negotiating spaces with the health system for claiming them. We note that community reflexivity, or in their own words 'feeling the pulse of the community’ was pivotal to prioritizing maternal health.

Incorporating reproductive and maternal health into the organizational cycle of the campaign reflects a strong community connect and reflexivity. Right to health campaign has continuously been in force for over 20 years, in distinct phases and with varied intensity, including joining forces with the civil society's national and state level campaigns. With JMS' overarching focus on addressing caste and gender-based violence against Dalit women for over 15 years, maternal health was not a priority issue for the collective. In the policy framework for implementation of the National Rural Health Mission (NRHM), the government of India included communitization as a crucial component [45] in which community-based monitoring and planning (CBMP) was adopted as a powerful method for community participation and feedback. JMS was part of the pilot phase of the communitization effort during 2008–10 in the district of Raichur. However, soon after the pilot phase, the official state-led CBMP was shelved, except in a few states, such as Maharashtra [18, 19]. Sporadic and disparate efforts to pursue the processes continued with the leadership of civil society in India elsewhere in India as well [46, 47]. The communitization intervention focused on reviving PHC services in general, institutionalizing community participation through the formation of village health and sanitation committees (VHSCs), now known as village health, sanitation and nutrition committees (VHSNC). It was not aimed at reproductive and maternal services alone [20]. Later, critical maternal health services, viz., ante-natal care (ANC) and post-natal care (PNC), gained significance with an emphasis on reducing maternal mortality. These service are organized in villages on a designated day every month where the front line health workers are mandated to be present, and are known as village health and nutrition day (VHND),

The beginnings of the maternal health rights campaign in the intervention area of JMS reflected such a reality which aptly explains the intensification of the campaign in varied evolving phases. During the year 2014–15, in the *karyakartha samiti* (the coordination committee) meetings, the attention of the collective was drawn to the episodic news of rampant referrals of pregnant women to private hospitals, and gradually, several stories of exorbitant expenditure for ANC, childbirth, neonatal care and PNC became known [JMS_MHFilm]. As they encountered an increasing number of instances of Dalit women suffering due to the exorbitant expenditures related to pregnancy and childbirth-related services, JMS *sanchalana samiti* responded to this emerging community issue with a decision to address this systematically.

*Health is our fundamental right. Because the PHCs do not function properly, Dalit women are the first to get affected and their rights violated. They are compelled to spend an exorbitant amount of money in private hospitals. Though we did community monitoring [of PHCs] earlier, now we thought it was imperative to take up this issue as JMS is a Dalit women's organization. We must focus on community issues* (Interview_SM1-Female, Pothnal, dt. 13 July 2020).

This period was marked by a regime change in the central government and several policy changes. Significantly, it marks the fading out of the NRHM that catalyzed the rejuvenation and functioning of rural health systems across India. Despite the NRHM, studies during this period showed that the 'hardship financing' for health care that included the selling of assets and out-of-pocket expenditure continued unabated in several critical areas of health care in rural India [48]. The health system needed continuous and intense policy interventions. However, the policy change around this period augmented the weakening of the public health care services and an associated marketization of health care, shifting towards an insurance-based service provisioning model [49, 50].

The understanding of reproductive and maternal health rights as integral to Dalit women's right to health and dignity found a natural and instant reception in the leadership of the women's collective. However, such a conviction gradually deepened in the community only through intense and prolonged discussions. JMS leaders recalled the evolution of the maternal health care campaign in the organization. They noted that though maternal health care was latent in the organization's overall right to health campaign, it evolved into a full-fledged campaign only when JMS took note of maternal health violations in the communities during 2014–15. (JMS-IGD_ 2020/4, Pothnal, dt. 13 August 2020).

### The SA process for maternal health rights

As discussed in this paper, the idea of ‘struggle’ in the three-pronged Ambedkarite approach is the motivational force that propels JMS into the practice of SA. In the following section, we delineate phases and the practice to understand the 'how' of the SA process for maternal health.

#### Articulating and phrasing issues in human rights language

Given the overarching human rights perspective, the collective swiftly formulated the articulation of maternal health services in the language of maternal health rights and, conversely, the non-availability or deficiencies of services as 'denials' or 'violations' of their rights. The human rights framework enunciates the components of availability, accessibility, affordability and quality of health care services as fundamental to the social arrangements of realizing the human right to health care [25]. Such an understanding resonates distinctly within the organization's overall strategic approach in articulating public services as entitlements and rights, and exclusion, non-availability and inaccessibility to public services as 'denial' and 'violation of human rights'. However, the seriousness attributed to the issue of maternal health is evident in how JMS incorporated this as a serious organizational strategy of a campaign.

#### Cyclical design and persistence

We find that the maternal health campaign that originated in the compelling issues raised by this Dalit women’s collective during 2014–2015 sustained itself over six years (2015–21) owing to its cyclical design, incremental value addition in gathering community evidence in each cycle and addressing newer issues as they emerged (see Table 3). The campaign process indicates that each cycle is marked by a value addition that has kept the campaign's momentum. Such value addition is seen in incorporating technical inputs and technology integration to systematize the evidence collection process.

Cycle one (2015 – 2016) laid the foundation for the campaign, where all the challenges faced by women during pregnancy and childbirth were documented. Such stories ranged from dysfunctional VHNDs where ANC services were supposed to be provided to stories of referrals to private hospitals during pregnancy and after childbirth. Building on this, during the second cycle (2017–2018), the CE process was made more systematic, incorporating visual tools for data collection. The visual tools were introduced for the CE process, some obtained from alliance partners such as NAMHHR. The refined and refurbished tool had sections that included information on the availability of maternal health services, adverse events such as maternal or child death, experiences of women who had childbirth regarding the quality of services and access to the benefits of schemes.

#### Building strategic alliances

Given the understanding of collective power, JMS has been part of several alliances, such as the right to food campaign, the people's health movement, and the wage-laborer's union. At the same time, learning from several campaigns, the organization incorporated and adapted newer methodologies and tools, enhancing its capacities in SA. It became a part of the National Alliance for Maternal Health and Human Rights (NAMHHR), a national-level network, in 2018. Through this alliance, they gained technical knowledge and access to a digital application, ‘*swasthy darparn,’* that NAMHHR developed to strengthen and streamline community monitoring of reproductive and maternal health services [51]. By 2018, the organization procured smartphones for the core leadership and trained them in using these phones. As a result, the neo-literate women leaders acquired new skills in navigating apps and using WhatsApp. During the third cycle (2019–2021), the CBO also became part of the Dalit Human Rights Forum – Karnataka (DHRF) and People's Forum for Justice and Health (PFJH) through which it was able to negotiate with the state health authorities.

Overall, the CE process indicates that it has organically evolved from anecdotal community experiences to making sense of the general situation of the deteriorating public health services. In terms of the organization's response, it has moved from ad-hoc negotiations with the local health care providers to a more systematic adoption of tools and incorporating an evidence-based approach, thus exhibiting tremendous adaptability by JMS. The design of the maternal health rights campaign reflected the cumulative organizational experience. It included mobilizing communities, building community perspectives, enabling leadership, and giving due attention to collecting evidence. In the analysis of the campaigns, we found a significant influence of the community processes of deepening the human rights consciousness around maternal health rights and strengthening community leadership through facilitating women leaders’ active engagement with health care providers using evidence.

### Deploying evidence in the SA process: dual role

Often report cards are projected as the ultimate outcomes in SA for health. In JMS, however, we found that the evidence generation and deployment process was grounded in the organization's overall ethos and central vision, viz., dignity and empowerment of Dalit women. The organization found creative ways to deploy the evidence in various forms to accomplish two-fold tasks, both horizontally and vertically. Horizontally, evidence and messages are used as a medium to mobilize the community further and to keep up the momentum. At the same time, it acquires a different role as a means of negotiation with the health and other administrative systems. The following section describes this dual role of the evidence.

#### Horizontal deployment of evidence for mobilization and empowerment

The forms of evidence generation, packaging of the evidence into several types of messages and the modes of deploying this evidence at the community level is marked by the elements of demystification of the evidence generation process, internalizing, and building ownership of the content. It is deployed in various modes to accentuate the momentum in the community for building human rights consciousness on maternal health rights.

##### Evidence generation and conscientization

In each campaign cycle, conscientization and community empowerment continued to be central to the evidence generation process. Preparing pictorial tools for the CE and administering them reinforced the awareness of maternal health entitlements. The protocols of maternal health services included in the tool referred to the periodicity, frequency, and location of availing these services. During the entire campaign, women were introduced to the pictorial tools to become versatile with the technical knowledge of ANC, PNC and PHC services and to articulate them as entitlements. Local language, including colloquial expressions, was used for discussions with key women leaders and workshops with the *karyakartha samiti.*

The women leaders conducted CE in each selected village of the 7 PHCs and used it as an opportunity to conscientize women members' human rights perspectives on maternal health, viz. Availability, accessibility, affordability and quality of services. The data were collated and converted into color-coded report cards in the local language. In the IGDs with the *sanchalana samiti*, women leaders unanimously confirmed it was a daunting task to understand the data initially. However, the color-coded pie charts placed with relevant images (pictures) and the mentors' discussions made it easy for them to comprehend and explain it to the community.

##### Community-oriented messages

The analyzed data were then converted into colorful report cards and innovative messages and were used to build on the community's momentum and continue the discussions with the members of the collective. The report cards for each PHC included color-coded pie charts for select indicators and were printed as large flex banners. The collated information was then converted into various communication materials for the community, such as wall-writing, pamphlets/leaflets in Kannada, posters, banners, songs, slogans, photo galleries, story scripts, and social media posts – WhatsApp, Facebook, Instagram and the website. More importantly, these were also converted into a memorandum with women's demands for submission to various authorities and press-release for the media.

##### Popular modes of presenting evidence

Horizontal-community level campaign was directed towards further mobilizing and empowering the communities by sharing the findings that alert them on the status of maternal health services, conditions of subcenters and PHCs. At the community level, the modes of deploying evidence have ranged from holding meetings in the communities along with the community health workers and public events such as village foot-marches (*padayathras*), and public hearings (*Jan samvad).*

Five key community-level activities mark such a process of intensifying the campaign in each of the villages of CE across all seven PHCs. (1) A preliminary meeting with the leaders of the village level unit of JMS to prepare for the community meeting of the entire village; (2) a public meeting on maternal health services in the village where the findings are presented on large banners and women leaders explain these findings while using it as the occasion to inform people on various maternal health services; (3) reading aloud a petition of demands; (4) collecting signatures on the memorandum representing the collective voice endorsing the demands therein; and, (5) finalizing a delegation of community members for memorandum submission and representation at the public health dialogue *(jan samvad)* to be held at the PHC.

JMS has optimally employed creative and imaginative use of public space for capturing the public imagination and building momentum for health system response.

The copies of the documents bearing signatures endorsing the memorandum, for example, indicated that cumulatively about six thousand people physically participated in these public events in each of the campaign cycles. The driving force for the community participation was the assurance that the issues would be taken up with the public authorities at various levels and the hope that a resolution to these grievances would be in place soon.

#### Vertical deployment of evidence for empowered negotiations

In the vertical deployment of data, however, the character of evidence presentation changes remarkably. We identified three noteworthy features that the neo-literate women's collective employed in disseminating the evidence to various constituencies, viz. packaging community evidence in diverse imaginative forms to attract attention of media, leveraging larger political spaces, and using sensitive -emotionally charged adverse events to transmit the evidence to the public authorities. This process aims to create the ambience for negotiations at the block and district levels. The official spaces include institutions and authorities comprising bureaucracy of health and other related departments such as Women and Child Development, elected representatives (viz. member of the legislative assembly -MLA and member of the legislative council—MLC), officials of Panchayati Raj Institutions (PRI), and creative use of print and visual media [52].

##### Petitions and charter of demands

The synthesized data is presented in several forms including petitions to various authorities, a charter of demands presented through brochures printed for public distribution, and press releases for the media. The content is presented as specific demands and claims for maternal health rights.

##### Leveraging public spaces and opportunities

The strategy of presenting evidence takes the shape of submitting memoranda, confronting the officials, interfacing with elected representatives, briefing media, public demonstrations in the form of public rallies, and the like.

##### Seizing the opportunities of political events

Leveraging political opportunities in the district stands out significantly where the community of women have been able to draw policy maker's attention to the local issues of maternal health care. Three significant electoral processes occurred during the maternal health rights campaign, viz. Karnataka State Legislative Assembly elections (12 May 2018), Chief Minister's village stay (23 July 2019), and GP elections (December 2020). These events are held in a politically charged atmosphere, and political representatives are on their best behavior with the citizens. Dalit women used these opportunities to make themselves heard on the issue of maternal health services by submitting the memoranda on maternal health demands – it included printing a People's Health Manifesto for maternal health care, reached it to the chief minister during his political event of village stay on 19 June 2019 [53]. While most of these are exercises of demanding public accountability having no instant tangible outcomes for the collective, JMS engages with these spaces to highlight people's issues. It also serves as a reference point for subsequent meetings with officials and in *gram sabhas* (Constitutionally mandated people’s assemblies at the ward level in a GP) [54].

##### The symbolic use of adverse events

Despite keeping a tab on the maternal health situation of fifty villages, women leaders admitted their chances of missing some adverse events for several reasons (JMS-IGD_2020/2, Pothnal, dt. 15 February 2020) [55–57]. The collective uses exceptional events as exemplars or symbolic evidence of the consequences of not meeting maternal health needs in villages. Community-level investigation of the maternal death of a young migrant woman who had come for delivery in one of the villages illustrates such an approach.

We noted that each event needed meticulous planning, a series of preparatory processes, and a relentless follow-up. Holding public health dialogues at PHCs (a pre-planned public meeting with the MO and health staff), holding rallies in the townships (Taluka or district headquarter), *dharnas* (sit-in protest by a large number of people or picketing) or holding press-conferences. The discussions with the leadership spelt out the purpose of such an intensive engagement in the process:

*Q: We have noted in many places that only the community is focused, not the authorities. Some other organizations focus only on meeting the authorities; can you tell us why you focus so much on both these directions?*

*A: We need many more people, especially women from Dalit communities, to be aware of this issue; hence, simplifying it in their language is critically important. Only then can we fight for the right to health. People are so busy with their work and wages (kelasa mattu kooli) that they open their eyes when adversities affect them.*

*If we go alone or in smaller numbers, authorities do not care. We need a greater number of people. At the same time, we need to reach this information to the authorities in diverse ways so that they will act.* When we call an MLA for the meeting, the DHO readily obliges us to come and listen (JMS-IGD_2020/1, Pothnal, dt. 08 February 2020).

Women leaders were clear on the purpose of such an interface. They expressed the need to build continuous pressure on the system to elicit responsiveness to the issues highlighted in the petitions so that maternal services are available and accessible to women. They also indicated that frequently, while negotiating at the higher level of administration, the community level functionaries start responding to the issues at the community level. However, the more complex demands placed at the district level may not be responded to immediately. A senior woman leader confirmed this in her own words:

*During our campaign, people raised the issue of not having an anganwadi center; hence, children, pregnant women, and young adolescents were not getting nutrition, as cooked food was not served in several villages. We gave petitions to GP Secretary. However, only when we put pressure on the deputy director of the women and child welfare department, Raichur, we got some response. The ICDS centres are now built, and an anganwadi worker is appointed.* **(**Interview_SM2-Female, Pothnal, 13 July 2020)

The analysis of the campaign reiterated that imaginatively packaging evidence, optimally synchronizing social media with the campaign, building capacity to harvest smartphone technology and creatively using public spaces have significant advantages to the campaign outcomes both in terms of building local momentum as well as the pace and quality of response by authorities. It could be considered one of the significant factors behind sustaining their campaign over a period. The conspicuous feature of this campaign was the way the leadership used the reports imaginatively to strengthen their collective power and to establish their negotiating power with the health system.

### Changes and challenges

Studies on accountability processes pursued in India in similar contexts of caste and gender show the possibility of ushering in political capabilities within communities, enhancing empowerment and the limitations of the systemic and tangible changes such processes can sustain [58, 59]. The persistent campaign by JMS and the changes experienced, too, confirm such an understanding. In this section, we present some of these changes in the overarching systemic human rights perspective of availability and accessibility [25] and the experience of empowerment Dalit women experienced as part of their dignity and social citizenship in the responsiveness they have experienced [23].

### Improvement in services

The SA process has resulted in considerable improvement in maternal health services. It is visible in the regularity of VHNDs and ANC services, positive attempts made by ANM and ASHAs to inform every household, and in the follow-up visits to those who could not attend VHND**.** Such a change is also seen in the ease that is introduced in accessing services such as ultrasound (sonography). Before this campaign, women were indiscriminately referred to private facilities at their own cost for such services. The leadership of the women's collective itself acknowledged that in some domains, these changes are incremental, and a more substantive change can be seen in some other services, such as ANC.

#### Enhanced responsiveness of authorities

JMS leadership regards the responsiveness of the administration and the elected representatives as a remarkable acknowledgment of their collective power. In the historical context of their disadvantages of being Dalit women, landless laborers and neo-literates, the hitherto unrelenting and unresponsive administration representing the upper caste hegemony now responds, acknowledges their grievances, and makes assurances to remedy the gaps. Dalit women regard this as the act of affirming their collective power. In the discussions with women, they were unanimous in affirming as follows:

*No one would even look at us earlier, leave alone listening to us. We were afraid to go to PHCs even for our health care. From that experience, over a period, due to our struggles through JMS, they regard us as leaders, are afraid to do discrimination, and they respond to our phone calls, petitions, and demonstrations. Now they know that we will not leave them. Earlier, even if we went and waited in front of their office, they would not listen to us. Whereas now we can get women admitted to hospitals just over a phone call.* (JMS-IGD_2020/4, Pothnal, dt. 13 August 2020).

These and many such narratives indicate increased responsiveness to Dalit women in the region by the local and district health authorities, leading to an enhanced sense of confidence in Dalit women to confront the duty bearers with information on the status of maternal health services. A senior woman leader said, ‘n*ow the Taluka Health Officer (THO) responds to our phone call and WhatsApp voice messages.’* (Interview_SM2-Female, Pothnal, 13 July 2020).

Dalit women leaders cited the example of a maternal death audit that was ordered in the district hospital affiliated with the medical college when JMS raised the issue of unaccounted maternal deaths in the district hospital [52, 55–57]. In addition, several official departmental orders were issued on several issues, such as the availability of medicines and instructions not to take money from patients, in response to its petitions (See Table 1). Some ASHAs belonging to Dalit communities found a great support in JMS’ campaigns and were appreciative of JMS’ intervention. They perceive JMS leaders as allies that have contributed to their profile on performance in ANC and PNC services. Some even had deeper insights into palpable changes in the relationship between people and health staff. One of the ASHAs from Balaganur PHC interviewed said:

*JMS has given us such rich information through pictures, pamphlets and banners. Pregnant women and mothers have immensely benefited from this. The officers fear the sanghatane. Because of this, corruption and instances of health staff demanding informal payments from people have reduced*. (ASHA-BLG*, Interview*, Balaganur, dt. 20 November 2020).

#### Impact during emergencies and COVID19 lock-down

The impact of such an enduring process of exerting pressure on the administration is seen in some adverse events and emergencies and was palpable during the stringent lock-down during the pandemic [60]. During the lock-down, staff absenteeism in all the PHCs on the pretext of not having a transport facility led to anxiety for all pregnant women. Women leaders immediately petitioned THO through WhatsApp voice message followed by a phone call. THO issued a circular on 21 April 2020, mandating the presence of all the staff in PHCs. [Resp.THO/2_2020].

Similarly, during the lock-down several public services in many villages, such as the supply of drinking water and distribution of food grains through Public Distribution System (PDS) shops, had dramatically stopped. The PDS owners refused to open shops, fearing the virus. The impact of Dalit women’s negotiations was experienced as they reached out to authorities over the phone and by sending WhatsApp messages, and a swift response was received [JMSChiguru_FB].

#### Enduring health service challenges

Despite the systematic processes of deploying evidence and investment of time and efforts of conscientizing the community, the cumulative outcomes of such a process, however, are not uniform. As the leadership acknowledged, the perception of the power of the collective by the authorities is not uniform across 7 PHCs. This was apparent in the differential participation and varied response of authorities to the local negotiations. Besides, women have acknowledged that certain areas of maternal health services, especially those entrenched in the nexus between the public health functionaries and private health care providers, were hard to break. Though the availability of ANC services was visible, the number of women delivering in private hospitals had also increased substantially. Meanwhile, women's out-of-pocket expenditure, both during pregnancy and for services during childbirth, also substantially increased during this period.

The community-based monitoring of maternal health services and a consistent interface with service providers is a demanding exercise needing constant vigilance. The responsiveness of the administration is not always positive or timely. We also noted that Dalit women leaders expressed such a process at times as a tiring, frustrating and futile exercise when the responsiveness is short-lived, untimely or hard to come by (JMS-IGD_2020/4, Pothnal, dt. 13 August 2020).

## Discussion: Dalit women, maternal health and SA

The growing literature on SA provides a sever scrutiny of SA methods and processes [22]. They do recognize that SA helps usher in improvement in specific public health care services [18]. However, there is also an acknowledgement that despite these efforts, due to the increased fragility of the health system, the disadvantaged communities continue to face challenges in accessing quality of care [61]. Given this context, in this section, we now revert to the vital question – how a group of neo-literate Dalit women internalized and contextualized their approach to SA to engage with the issues of their maternal health? What insights do their approach and processes offer to the practice of SA?

### The lens of caste to unpack layers of social exclusion

Though disaggregated data by caste is not available on maternal health services, various accounts indicate that Dalit women belonging invariably to the lower socio-economic strata have marginal negotiation power. Their challenges in accessing maternal health care services tend to be more acute than other women [62, 63]. The story of JMS reveals that by adopting the perspective of caste as the axis of marginalization, it has been able to unpack the systemic processes that affect Dalit women. More significantly, the political understanding of the women's collective reveals that the challenges in maternal health are not isolated from their life-conditions, where they face discrimination in every sphere of their life.

The organization identified the caste-based discrimination and social exclusion that translates into the cultural norm of 'physical segregation' as having significant bearing on Dalit women accessing reproductive and maternal health services. Such a diagnosis has led them to pursue pathways to address such issues that go beyond advocating for health services alone. It entails centre-staging dignity as the fulcrum of realizing citizenship and is fundamental to the process of accessing services.

Therefore, their demand for accountability goes beyond the confines of limited services. Research too strongly suggests that for marginalized communities, along with demanding availability of institutional services, acquiring the power of negotiations is of stellar importance [64]. Theoretical perspectives on accountability practices and employing them strategically have accounted for augmenting marginalized community's power of negotiations [22]. The analysis in this paper offers valuable insights towards accomplishing this, which also necessitates going beyond the traditional SA frameworks.

### Conscientize-organize-struggle: Ambedkarite approach to SA

This paper exhibits the pursuit of a grass-roots Dalit women's collective in claiming dignity, which forms the fulcrum of its accountability strategy. Addressing indignity that Dalit women face based on their caste, gender and class, is central to such a strategy. Their experience of social exclusion from public spaces and institutions owing to their caste, is compounded by toxic prejudices that confront them based on gender and class (as women agricultural laborers). Against this backdrop, the sense of dignity and empowerment they feel now as part of JMS encapsulates the essence of their collective struggle and is a testimony to the outcomes of human rights-based SA. Therefore, the analysis in this paper foregrounds human rights conscientization, and not merely accessing a few services, as central to claiming dignity. In the JMS campaigns, as illustrated in the maternal health campaign, we find the imprint of human rights consciousness as the overarching canvas for demanding accountability for health and wellbeing.

Such an understanding and the strategy of claiming dignity is firmly woven around the three-pronged strategy of Dr B. R. Ambedkar, viz. conscientize – organize and struggle. While Dalit movements have used this as part of their strategy primarily in the civil and political rights domain, the paper demonstrates that the Ambedkarite strategy could potentially re-articulate the meaning and approach of SA strategies while addressing the issues of the most marginalized communities in India. JMS used this three-pronged strategic approach to address issues of social exclusion and discrimination for over two decades. As the challenges of maternal health emerged, the organization swiftly rode on this mobilization and adopted the same approach to address the issue of maternal health﻿ rights violations.

### Privileging SA process over tools and methods

In contrast to the depiction of SA as a set of sequential or linear events or tasks, its approach needs to be fore-fronted as a continuous, persistent, iterative, multi-level and cyclical process within which participation and empowerment are implanted. The SA discussed in this paper in the context of maternal health alludes to such an understanding. It combines the bi-directional momentum, viz. horizontal and vertical engagement, and stimulates the outcomes of empowerment and accountability. The community of women leaders engage persistently with the processes of conceptualization, collection of community evidence, deciphering the results, and innovation on ways to deploy data for collective empowerment. The leadership leverages these processes horizontally to empower the village-level leadership of JMS. Processed information, with some technical support from mentors in analyzing, decoding and strategizing, is deployed vertically to elicit public commitment and responsiveness. The intended duty bearers are the health bureaucracy, political leadership and other intersecting power-wielders in the domains of social determinants of health, viz. water, sanitation, food and nutrition.

We need to move beyond the naive assumption that collated data represented through report cards as evidence in itself has the potential for ushering in change in maternal health services. The analysis in this paper reiterates that strategic deployment of the evidence is of paramount importance, especially when the community is deemed to be powerless. Such deployment of evidence needs to be done in multiple forms and at various levels of the political and administrative hierarchy to enhance the negotiating power of marginalized communities. The women’s collective employed a range of strategies spanning from familiar public dialogues and conventional submission of petitions to more aggressive press conferences, television interviews, and sit-in protests demanding response and tangible action. The voice strengthened and articulated at the horizontal levels with the communities adds teeth to such vertical actions. The evidence gathered systematically and processed contextually provides an edge to compelling responsiveness and corrective actions from duty bearers located both within the health services system and outside﻿ in the socio-political arena. The evidence-based SA indicates to having a significant bearing on bringing the duty-bearers to the negotiating table with the Dalit women agricultural laborers. Deploying evidence in multiple ways, community empowerment, building community discourses, and exerting public pressure at the local and higher levels of the health care system have cumulatively contributed to tilting the balance of power in favor of Dalit women.

### Potential of human rights perspective for bolstering SA

Conceiving SA as a strategic approach, rather than merely being treated as a method, and integrating it with human rights perspective, has the potential to galvanize social citizenship. It opens ways to identify violations of the human right to health in spaces and institutions based on gender and caste. Social rights offer the premise and logic to consolidate and strengthen social citizenship [23]. JMS' campaign for maternal health rights exemplifies the process of claiming space and dignity, an essential part of citizenship, through seeking accountability in maternal health care.

The agency and ability of local women's leadership to articulate issues in a human rights language, regardless of their educational and literacy status, emerges as pivotal to SA, as this paper indicates. Dalit women spearheaded the mobilization processes and functioned as critical presenters and anchors of the public health dialogue at the PHCs that saw the presence of MOs, nurses, ASHAs, VHSNC members, GP members and other health staff. This is a significant shift of power, as until recently in India, non-Dalits did not even see the shadow of Dalits, leave alone hear them or sit together. While it served as an education process for everyone, it also consolidated Dalit women's leadership, now confident with new knowledge of health policy and programme idioms, that included maternal health entitlements. Since they owned and led the entire process, and the report cards were pictorially displayed, they needed no prompting or rehearsal in presenting the data and findings. Invariably, across the seven PHCs, the MOs﻿ neither had excuses about the evidence presented﻿ nor did they exhibit any resistence, as it was not presented as a fault-finding process. The autonomy of the organization and the fact that the compulsions of funded project cycles did not prompt these actions, allowed the CBO the liberty and flexibility to customize it to the local contexts, cultural idioms and pace.

The cumulative impact of the CE relentlessly pursued through the human rights framework of a campaign points to positive gains in domains beyond the maternal health services, including ICDS and other health care services in PHCs. These gains are available to all the communities, including those that were not part of the campaign.

The significant question that arises then is about the sustainability of the SA process. As seen in JMS, the initiation of SA was to address the underlying structural determinants of health, viz., social exclusion. Hence, within their intensive quest for claiming dignity and justice, reproductive and maternal health became added spaces for realizing social citizenship. Broadly, it indicates that the strategic location of SA for maternal health within the organizational paradigm is of paramount importance. If such a paradigm is imbued with human rights perspectives, the SA process for maternal health will be of considerable depth. This insight from the story of this grass-roots Dalit women’s collective adds an essential but nuanced facet to the idea of 'strategic' deployment of SA processes compared to considering it merely as a 'tactical’ problem-solving tool [22]. Sustaining such an intense SA process or the changes in health services, therefore, cannot be construed as the result of interventions in health system alone.

## Conclusion

SA practices and processes are accepted as complex processes [21, 65]. Such complexity is rooted not only in the health system alone but also results from the graded inequality embedded in these institutions. The marginalized community experiences graded inequality as unequal social power, social exclusion, discrimination and indignity based on the axes of marginalization. The case of Dalit women agricultural laborers exemplifies this through the triple burden of inequality they experience based on caste, patriarchy and class. Considering this, SA for such communities is not only a technical process but primarily a political one. Hence, in addition to the attention and rigour required in the technicality of generating evidence, the approach to SA and evidence deployment is quite crucial to address the structural power inequities that birth and sustain violations of health and other social rights. We see this analysis in the paper as contributing to foregrounding community power and the processes of building counter-vailing power of community empowerment that are central to sustaining SA not merely as tools of community participation but as pathways of empowerment and claiming citizenship.

In India, as the private and corporate health care sector has grown phenomenally, and therefore has implications to employing the human rights framework for SA. As the power shifts from the state to non-state actors, the possibility of engaging the human rights framework becomes bleak. There is a compelling need for expanding the human rights framework to bring the non-state actors and private domains into its ambit. The regulatory vacuum for the private-corporate sector in countries like India provides ample scope for compromising the human rights gains in health and the social citizenship that disadvantaged communities have experienced.

Growing academic literature has made significant contributions toward conceptualizing SA. The findings of this paper might be of greater interest to the practitioners of SA in general and those focus on health, which includes human rights advocates and researchers aiming to strengthen public health, health care, and maternal health. Additionally, researchers on accountability would gain from the insights of this paper to explore questions such as 'what does SA mean in practice in a particular context, 'what are the mechanisms and processes of SA that bring and sustain change to a marginalized community?', 'how can SA be deployed as a strategic political tool to address the systemic discrimination that perpetuates marginalization?'. Such questions arising from this paper open potential areas for deeper explorations in the dynamic field of SA practice.

## Data Availability

No datasets are generated as part of this research study. The primary data that forms a small component of this study are interview summaries and transcripts. These data cannot be made available to safeguard the confidentiality and anonymity of the respondents. The secondary data, comprising a major source of this research includes digital data available in the public domain and the archival documents. For the former, appropriate digital links are made available in the paper. The latter are accessed by authors with due permission from the organisation only for the purpose of writing this paper. They are in the official language of the state of Karnataka, viz. Kannada, and are in hard copies. These are available at the organization at a reasonable request.

## Abbreviations

ANC: Ante-natal care
ASHA: Accredited social health activist
CBMP: Community-based monitoring and planning
CBO: Community-based organization
CE: Community enquiry
DHRF: Dalit Human Rights Forum – Karnataka
GP: Gram Panchayat
ICDS: Integrated child development scheme
ICESCR: International covenant on economic, social and cultural rights
IGD: In-depth group discussion
JMS: Jagrutha Mahila Sanghatane
KII: Key informant interview
MLA: Member of the legislative assembly
MLC: Member of the legislative council
MO: Medical officer
NAMHHR: National Alliance for Maternal Health and Human Rights
NRHM: National rural health mission
PFJH: People's Forum for Justice and Health
PHC: Primary health centre
PNC: Post-natal care
PRI: Panchayati raj institution
SA: Social accountability
THO: Taluka health officer
VHND: Village health and nutrition day
VHSNC: Village health, sanitation and nutrition committee
